# Radiologic-pathologic autopsy correlation of an internal watershed infarct, a case report

**DOI:** 10.4322/acr.2023.448

**Published:** 2023-10-23

**Authors:** Thomas Dimitrios Zaikos, David M. Yousem, Juan C. Troncoso, James Stephen Nix

**Affiliations:** 1 The Johns Hopkins Hospital, Department of Pathology, Division of Neuropathology, Baltimore, Maryland, USA; 2 The Johns Hopkins Hospital, Department of Radiology, Division of Neuroradiology, Baltimore, Maryland, USA; 3 University of Arkansas for Medical Sciences, Department of Pathology, Little Rock, Arkansas, USA

**Keywords:** Internal watershed, infarction, autopsy, neuropathology

## Abstract

Internal watershed infarcts (IWIs) occur at the junction of the deep and superficial perforating arterial branches of the cerebrum. Despite documentation in the radiology literature, IWIs are rarely encountered at the time of autopsy. Here, we report the case of a 59-year-old incarcerated male who was brought to the emergency department after being found unresponsive on the floor of his jail cell. Initial examination and imaging demonstrated right-sided hemiplegia, aphasia, right facial droop, and severe stenosis of the left middle cerebral artery, respectively. Repeat imaging 4 days after admission and 26 days before death demonstrated advanced stenosis of the intracranial, communicating segment of the right internal carotid artery, a large acute infarct in the right posterior cerebral artery territory, and bilateral deep white matter ischemic changes with a right-sided “rosary-like” pattern of injury that is typical of IWIs. Postmortem gross examination showed that the right deep white matter lesion had progressed to a confluent, “cigar-shaped” subacute IWI involving the right corona radiata. This is the first well-documented case of an IWI with radiologic imaging and photographic gross pathology correlation. This case uniquely highlights a rarely encountered lesion at the time of autopsy and provides an excellent visual representation of internal watershed neuroanatomy.

## INTRODUCTION

Watershed, or border-zone, infarcts are manifestations of hypoxic-ischemic injury within vulnerable brain areas that lie between major vascular supplies and are thus susceptible to hemodynamic insufficiency. Although watershed infarcts classically encountered at autopsy involve superficial cortical areas, such as border-zones between the anterior cerebral artery (ACA), middle cerebral artery (MCA), and posterior cerebral artery (PCA), internal watershed infarcts (IWIs) occur in the corona radiata or the centrum semiovale^1^ white matter structures located between the lateral ventricles and superficial neocortex. Specifically, the internal watershed region occurs at the junction of the vascular supplies between the superficial perforating arteries of the ACA, MCA, and PCA and of the deep perforating arteries, such as the lenticulostriate arteries, Heubner artery, and anterior choroidal arteries.^2-4^

Though rarely encountered in neuro-autopsy practice, internal watershed pathology is well-described in the neuroradiology literature. In the acute phase, IWIs appear as a chain, or beaded “rosary-like” pattern, of ischemia in the deep white matter of the centrum semiovale or corona radiata. Persistent hypoxic-ischemic physiology can lead to the development of a confluency of injured tissue that may be appreciated in a “cigar-like” pattern.^4-5^

This case report presents the first well-documented IWI with both autopsy and neuroradiology findings. The case provides a post-mortem correlation to radiologic evidence of disease, and illustrates the internal watershed zone by gross anatomy.

## CASE REPORT

The patient was a 59-year-old incarcerated man with a past medical history of hypertension, insulin-dependent diabetes mellitus, ethanol use disorder, and schizophrenia. He was brought to the hospital after being found down and unresponsive in his jail cell. Initial clinical evaluation was remarkable for right-sided hemiplegia, aphasia, and right facial droop. Head computed tomography angiography revealed occlusion of the M2 (Sylvian) segment of the left MCA. Mechanical thrombectomy was performed with complete reperfusion of the affected brain parenchyma. The patient also incidentally tested positive for SARS-CoV-2 nucleic acid by nasal swab.

**Figure 1:**
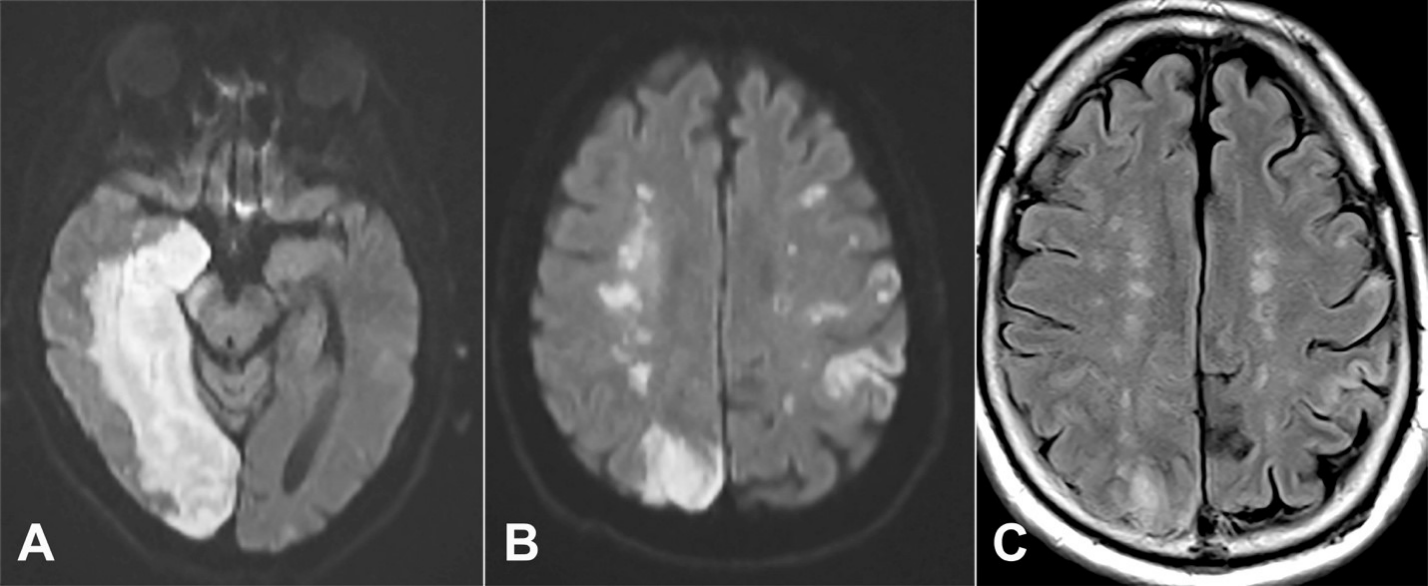
Magnetic resonance imaging from hospital day 4. **A -** the acute large right posterior cerebral artery infraction is seen affecting the right medial temporal lobe and occipital lobe on the diffusion weighted imaging; **B -** at the upper border of the right parieto-occipital cortical infarction, one can see a string of bright areas in the deep centrum semiovale on the right side representing acute watershed infarction on this diffusion weighted image. Acute cortical strokes are seen in the left hemisphere; **C -** the fluid attenuated inversion recovery (FLAIR) scan confirms the deep watershed infarctions bilaterally, seen as higher signal intensity on this imaging. The visualization on FLAIR suggests the infarctions are over 6 hours old. Diffusion weighted imaging remain positive for 7-15 days.

Four days later, the patient developed a new right hemispheric disease and was found to have a new large acute infarct of the right posterior cerebral artery territory (Figure 1). Bilateral deep white matter ischemic changes were also identified with a beaded, “rosary-like” pattern tracking along the internal watershed zone of the right corona radiata in extension from the adjacent PCA infarct (Figure 1). Despite medical intervention, the patient died approximately a month following admission.

**Figure 2:**
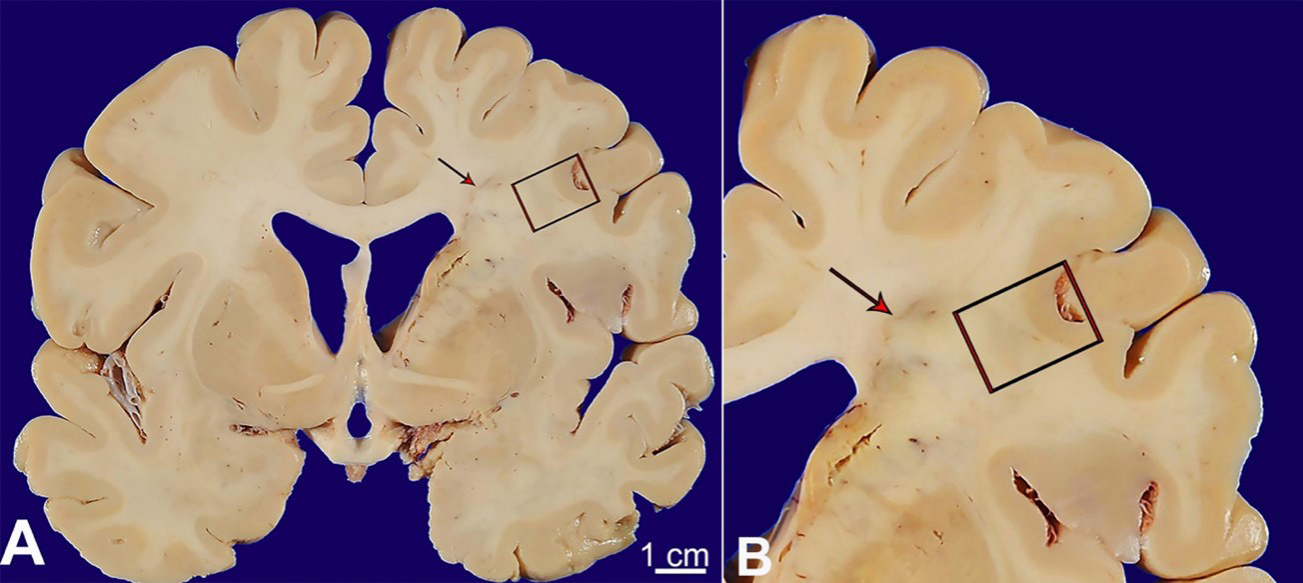
**A** and **B -** gross and microscopic pathology of internal watershed infarct; **A -** a confluent, “cigar-shaped” lesion involving the right hemispheric deep white matter was identified at the time of brain cutting (red arrow). This lesion extended from the frontal pole to the parieto-occipital lobe.

**Figure 3:**
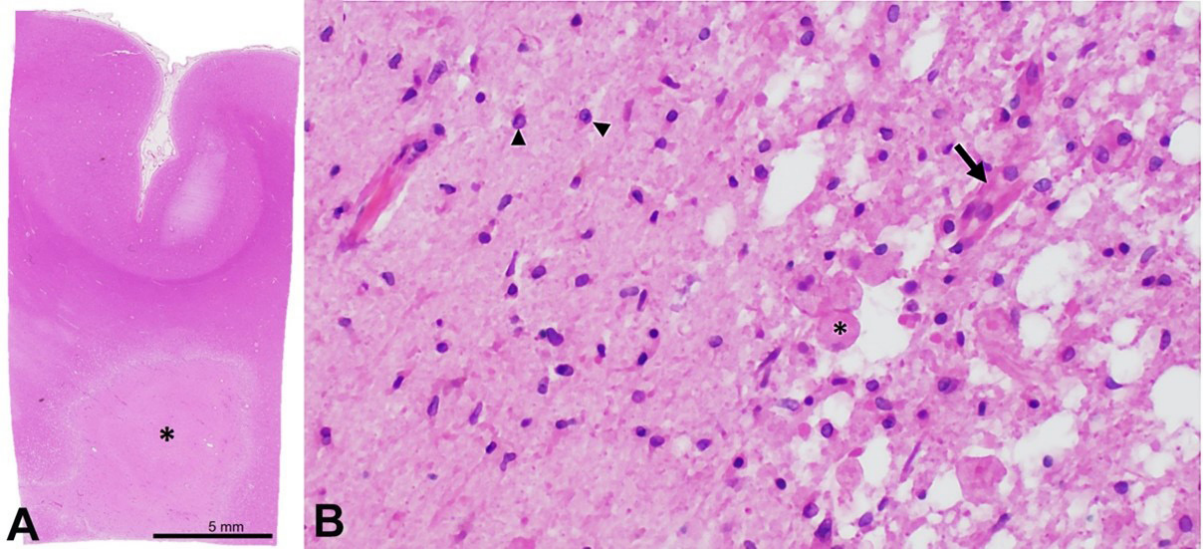
Microphotographs of the Brain - **A -** low-magnification photograph of representative section of the lesion (asterisk) and overlying cortex (section taken from area of red box from panel A); **B -** representative histology of the interface between the lesion demonstrating a necrotic core with infiltrating macrophages (asterisk) and neovascularization (arrow) as well as adjacent reactive glial tissue (arrowheads) (**A -** H&E; **B -** H&E, 200x).

External examination of the brain and attached vessels revealed multifocal moderate to severe atherosclerosis, including severe disease in the communicating segment of the right internal carotid artery with approximately 90% stenosis. Serial coronal sections revealed multiple lesions scattered throughout the cerebral hemispheres concerning acute/subacute to chronic infarcts. The largest involved the caudal right hemispheric deep grey matter and medial temporal lobe, extended to the right occipital pole, including the calcarine sulcus and visual cortex and corresponded to the PCA territory infarct identified on antemortem imaging. Adjacent to, but not contiguous with the large PCA infarct, was a separate, well-demarcated wedge/”cigar-shaped” lesion involving the right deep white matter, extending from the frontal pole to the parieto-occipital lobe (Figure 2). Hematoxylin and eosin-stained tissue sections from this second lesion revealed a subacute infarct with central necrotic debris, neuronal loss, rarefaction, macrophage infiltration, neovascularization, axonal spheroids, and gliosis (Figure 3).

## DISCUSSION

Internal watershed infarcts are a relatively common clinical, physiologic consequence of hypoxic-ischemic injury to a specific vulnerable region of the deep white matter, representing approximately 37% of acute ischemic events.^6^ In isolation, IWIs are rarely fatal; however, they are associated with increased morbidity compared to cortical watershed infarcts alone, which is likely associated with their incidence being a marker of significant large vessel cerebral hemodynamic instability and cortical infarction. As such, the identification of IWIs both at the time of neuroimaging and at the time of autopsy may be confounded by subsequent superimposed large vessel infarct pathology.^7^

The case presented here provides radiologic and post-mortem documentation of an evolving IWI. Similarly to the patient’s reported clinical history, cardiovascular disease, and proximal carotid atherosclerotic disease are important risk factors for the development of internal watershed infarcts in addition to diabetes mellitus; however, smoking and hypertension have not been independently associated with IWI when compared to general stroke patients in one retrospective study.^1^ The pathogenesis of IWIs is thought to be secondary to hemodynamic instability and severe hypoperfusion,^4-5^ and the current findings support this hypothesis, showing bilateral white matter ischemic changes as well as right hemispheric IWI extension in the context of hemodynamic compromise secondary to a large PCA infarct. With respect to the patient’s incidental diagnosis of asymptomatic COVID-19, it is unclear whether this had any significant impact on his risk of ischemic stroke. Although some COVID-19 patients may suffer from hypercoagulability due to some, as yet, unknown mechanism, the incidence of ischemic stroke in COVID-19 patients is only about 2-3%, and most of these patients have conventional vascular risk factors, and mechanism of ischemic injury.^8-9^ Finally, the timeline of the development of a beaded pattern of IWI on radiographs four days post-admission, followed by the identification of a “cigar-shaped” IWI at autopsy nearly a month later, is supportive of the “rosary-like” pattern of IWI progressing to a confluent pattern over twenty-two days.

In addition to providing a unique pathologic-radiologic IWI, the case is instructive to autopsy practitioners. While superficial cortical watershed infarcts are well-known in autopsy pathology and major superficial watersheds are routinely sectioned at the time of brain cutting, the internal watershed is rarely a consideration at the time of autopsy. Failure to recognize the neuroanatomy corresponding to the internal watershed could lead to diagnostic confusion, such considering different, erroneous entities that may include demyelinating diseases or superimposed infections. Recognizing the pattern of IWIs facilitates accurate and succinct pathology correlation within the clinical context of hemodynamic instability and evolving ischemic insult.

No published example of a gross correlate to IWI was found on literature review using relevant search criteria pertaining to gross, histological, pathological, or neuropathological correlates to radiologically and clinically documented IWIs, and for this reason, the case stands as a helpful demonstration of this pattern of infarct for autopsy practitioners. The only major limitation of this case report is that only a single example of a well-documented gross representation of an IWI is reported.

## CONCLUSION

In conclusion, this case report provides the first well-documented gross autopsy correlation to an evolving IWI, an ischemic lesion that arises in the white matter border-zone between superficial and deep perforating arteries. The progression of IWIs from the early stage, “rosary-like” pattern, to a later stage, confluent, “cigar-shaped” lesions, occurred over approximately 22 days. Understanding of the clinical context, premortem neuroradiology (if any), and gross neuroanatomy is important for the identification and accurate post-mortem diagnosis by autopsy practitioners of this infarct pattern.
